# Differences in spine growth potential for Sanders maturation stages 7A and 7B have implications for treatment of idiopathic scoliosis

**DOI:** 10.1007/s43390-024-00829-8

**Published:** 2024-02-19

**Authors:** Yusuke Hori, Burak Kaymaz, Luiz Carlos Almeida da Silva, Kenneth J. Rogers, Petya K. Yorgova, Peter G. Gabos, Suken A. Shah

**Affiliations:** Department of Orthopaedic Surgery, Nemours Children’s Health, 1600 Rockland Road, Wilmington, DE 19803 USA

**Keywords:** Brace treatment, Growth cessation, Height velocity, Idiopathic scoliosis, Skeletal maturity

## Abstract

**Purpose:**

This study aimed to clarify the differences in spine and total body height growth and curve progression between Sanders maturation stage (SMS) 7A and 7B in patients with adolescent idiopathic scoliosis (AIS).

**Methods:**

This retrospective case–control study involving patients with AIS at SMS 7 evaluated the differential gains in the spine (T1-S1) and total body height and curve progression between SMS 7A and 7B. A validated formula was used to calculate the corrected height, accounting for height loss due to scoliosis. A multivariable non-linear and logistic regression model was applied to assess the distinct growth and curve progression patterns between the SMS 7 subtypes, adjusting for potential confounders.

**Results:**

A total of 231 AIS patients (83% girls, mean age 13.9 ± 1.2 years) were included, with follow-up averaging 3.0 years. Patients at SMS 7A exhibited larger gains in spine height (9.9 mm vs. 6.3 mm) and total body height (19.8 mm vs. 13.4 mm) compared with SMS 7B. These findings remained consistent even after adjustments for curve magnitude. Non-linear regression models showed continued spine and total body height increases plateauing after 2 years, significantly greater in SMS 7A. More SMS 7A patients had curve progression over 10°, with an adjusted odds ratio of 3.31.

**Conclusion:**

This study revealed that patients staged SMS 7A exhibited more spine and total body growth and a greater incidence of substantial curve progression than those at 7B. These findings imply that delaying brace discontinuation until reaching 7B could be beneficial, particularly for those with larger curves.

**Level of evidence:**

Level III (Case–control study).

## Introduction

Adolescent idiopathic scoliosis (AIS) is a spinal deformity that primarily progresses during the growth spurt in adolescence [1]. Accurate assessment of bone maturity is crucial for managing AIS, as the likelihood of curve progression is closely related to a patient’s growth potential [2]. Traditionally, the Risser grading system [3] has been used to determine skeletal maturity; however, its significant variability has led researchers to explore alternative methods [4–6]. The Sanders skeletal maturity staging system has emerged as a more accurate and reliable marker of growth and curve progression [7, 8]. The Sanders maturation stage (SMS), derived from the Tanner–Whitehouse III method [9], simplifies the assessment of bone maturity and is widely adopted in AIS treatment due to its high reliability and ease of use [10–16].

The early mature stage, known as SMS 7, represents patients near full skeletal maturity [8]. It is believed that reaching SMS 7 marks the cessation of spinal growth and that minimal curve progression should occur. Nevertheless, Grothaus et al. [17] reported that some female patients with AIS who reached SMS 7 still experienced curve progression, with a subset requiring surgery or experiencing curve progression of 50° or more. This observation underlines the need for a deeper understanding of growth potential and curve progression in patients at SMS 7.

To address this issue, Cheung et al. [18] proposed a subclassification of SMS 7 into 7A and 7B based on the stages of ulnar physeal closure. Their findings suggest that brace weaning at SMS 7B allows for better prediction of curve progression and enables brace discontinuation without increased risk of curve progression before a patient reaches skeletal maturity. No studies have accurately evaluated the growth potential of the spine or total body in SMS 7A and 7B. This study aimed to clarify the differences in spine and total body height growth between SMS 7A and 7B, as well as to examine curve progression in both subtypes.

## Methods

### Study population

In this single-center, retrospective cohort study, we examined patients diagnosed with idiopathic scoliosis (ICD-10 code: M41.11 or M41.12) and classified as SMS 7 at our facility between January 2012 and December 2021. The study was approved by our Institutional Review Board. Eligible patients were required to have hand radiographs and posteroanterior views of EOS 2-dimensional (2D) imaging performed concurrently at the baseline visit, total height measurements, and EOS imaging of their full spine at both the baseline and all follow-up visits. To be included, patients needed to be followed until skeletal maturity, defined as Risser grade 5, or for at least 2 years of observation since SMS 7 diagnosis, considering the difference in bone age between SMS 7 and 8 [8]. Exclusions were made for patients with non-idiopathic scoliosis, whole spine radiographs instead of EOS imaging, insufficient hand radiographs for distal ulna assessment, or a curve exceeding 50° at the baseline visit.

Of the initial 1248 patients reviewed, exclusions were made for 22 patients with other diagnoses potentially causing scoliosis (non-AIS), 87 with whole spine radiographs instead of EOS imaging, 7 without concurrent hand radiographs with EOS imaging, 32 with inadequate hand radiographs for distal ulna epiphyseal status evaluation, 28 with a diagnosis other than SMS 7, 46 with a curve of 50° or more, and 795 patients lacking follow-up.

### Demographic data

Patient demographics such as age, sex, race, ethnicity, body mass index, menarche date, and visit date were collected by reviewing the charts. In addition, the Risser stage (0–5) and curve pattern (thoracic, thoracolumbar/lumbar, or double major) were assessed with a posteroanterior EOS 2D image.

### Assessment of hand radiographs

**Fig. 1:**
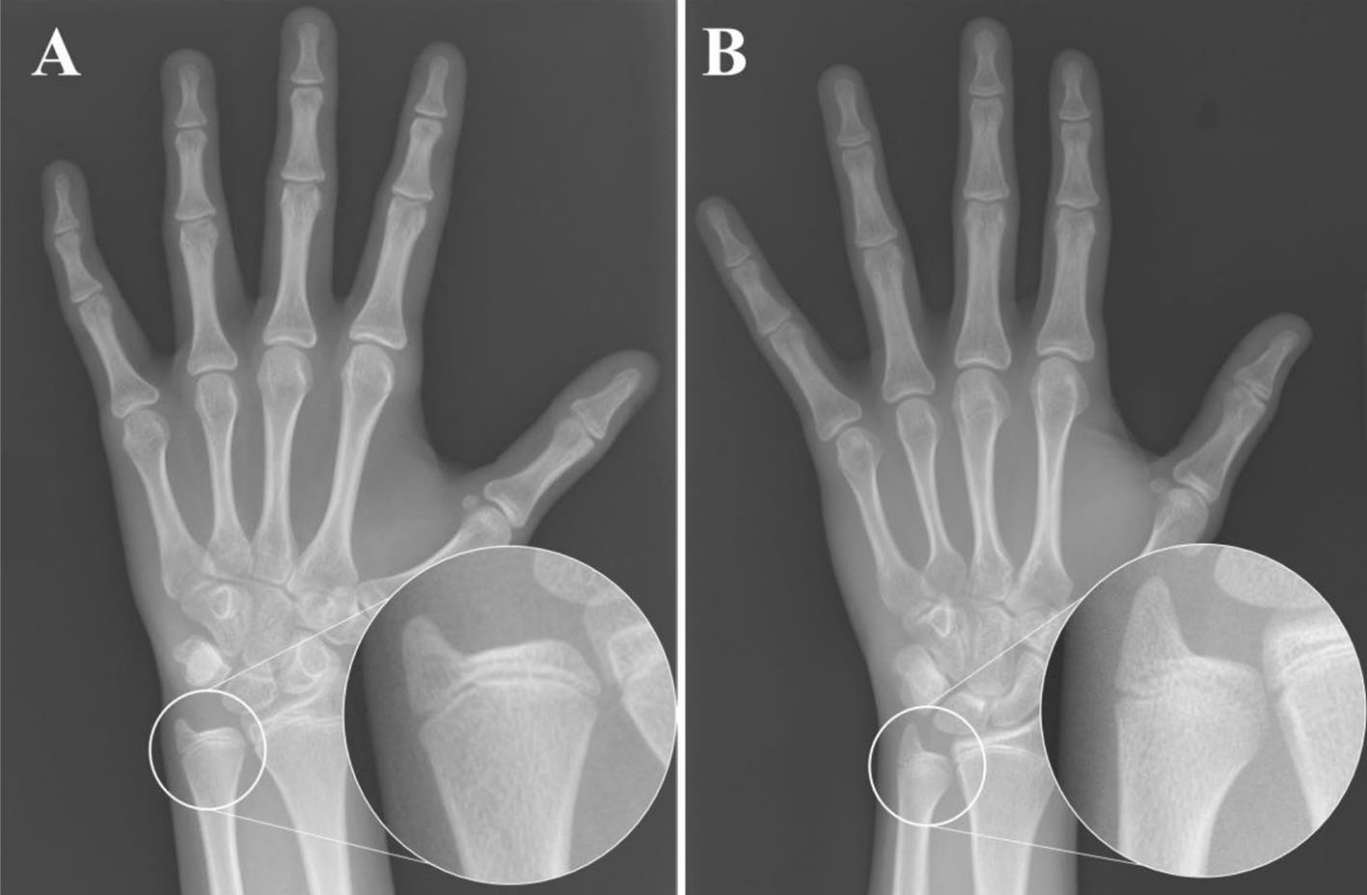
Radiographs of hands classified as Sanders maturation stage (SMS) 7A and 7B. In SMS 7A, all phalangeal physes are fused and only the distal radial physis is open with narrowing of the medial physeal plate of the distal ulna (**A**). In SMS 7B, there is fusion of > 50% of the medial growth plate of the distal ulna (**B**)

All patients had hand radiographs, which were assessed by physicians for SMS 7 in daily practice but were not subtyped into A or B, at the baseline visit. Two raters independently reviewed hand radiographs and classified them into SMS 7A, in which all phalangeal physes are fused and only the distal radial physis is open with narrowing of the medial physeal plate of the distal ulna (Fig. 1A), and 7B, in which fusion of > 50% of the medial growth plate of the distal ulna exists (Fig. 1B) [18]. We also referred to ulnar grading of the distal radius and ulnar classification [17]. Radiographs that could not evaluate the distal ulnar epiphyseal nucleus due to improper incident angle or rotation of ulna were excluded.

### Outcome measurement

To measure the magnitudes of major and minor curves, a posteroanterior view of EOS 2D imaging was utilized employing the Cobb method. Only EOS imaging was eligible for accurate calibrated length measurements, thus whole spine radiographs were not considered. Spine height was gauged as the distance between the centers of the superior endplates of T1 to S1. Patients undergoing brace treatment were instructed to remove their braces about 24 h prior to the visit, with measurements taken on brace-free radiographs. Total body height was gathered from chart reviews from the same visit, measured by clinic medical assistants in a standardized manner. Given scoliosis can cause height loss, we calculated the “corrected” spine and total body heights by adding the measured heights to the estimated loss of height derived from Stokes’ formula [19]. This formula is given as 1.55 − 0.0471Cobb + 0.009Cobb^2^ for a single curve and 1.0 + 0.066Cobb + 0.0084Cobb^2^ for double curves [19, 20]. Height gains and major curve progressions were then calculated from baseline visit to the final follow-up visit.

### Brace treatment

Information regarding brace treatment was retrospectively collected from the patients’ medical records. When patients reached skeletal maturity, discontinuation of bracing was considered. Our criteria for determining cessation of growth included stable height for 6 months, 2 years since menarche, fully capped Risser 4, or SMS 7. The decision to discontinue the brace entirely, gradually wean the patient off the brace, or continue bracing was made on an individual basis by the treating physicians. Factors such as the patient’s degree of skeletal maturity, curve magnitude, curve type, and the rate of curve progression were comprehensively considered in determining the most appropriate course of action.

### Statistical analysis

Continuous variables were expressed as mean ± standard deviation (SD) and categorical ones as numbers (percentages). For continuous variables, the *t*-test was used if normally distributed; otherwise, the Mann–Whitney *U* test was applied. The chi-squared test or Fisher’s exact test addressed categorical variables. We assessed inter- and intra-observer reliability using the Cohen’s κ coefficient. To explore associations between SMS 7 subtypes, height growth trajectory, and curve progression, a multivariate non-linear regression model was used utilizing longitudinal data. Restricted cubic splines with three knots assessed non-linear relationships between time, height, and curve magnitude. An interaction between SMS 7 subtypes and months was observed. Robust standard errors were computed to account for clustering of repeated measurements within individuals. A multivariate logistic regression model further assessed the influence of SMS 7 subtypes on significant curve progression. Our multivariate models were rigorously adjusted for confounders. All statistical tests were performed with a two-sided significance level. A *P* value < 0.05 was statistically significant, and all computations utilized R statistical software [21].

## Results

This study included 231 participants (191 girls and 40 boys), with a mean curve magnitude of 32.6 ± 8.0°. The girls had an average age of 14.0 ± 1.0 years, while the boys were older with an average age of 15.6 ± 1.0 years. By the final follow-up, girls were 17.0 ± 1.8 years and boys 17.9 ± 1.4 years with an average 3.0 ± 1.4 years since SMS 7 identification. At this point, 77% of SMS 7A and 84% of SMS 7B reached Risser stage 5, while the remainder were fully capped at stage 4. There was a substantial agreement in identifying SMS 7A or 7B with inter-rater reliability at *κ* = 0.74 (95% CI 0.64–0.84) and intra-rater reliability at *κ* = 0.80 (95% CI 0.71–0.88). Demographics showed that patients at SMS 7A were younger and had shorter total body height than those at 7B (Table 1). Baseline curve characteristics were comparable between 2 subtypes (Table 2).

**Table 1:** Demographics

	SMS 7A	SMS 7B	*P* value
Age, mean (SD)	14.1 (1.1)	14.6 (1.1)	< 0.001
Sex, girls (%)	128 (86)	73 (78)	0.092
Months from menarche, mean (SD)	14.5 (6.5)	19.0 (12.0)	0.014
Race, *n* (%)			
White/Caucasian	108 (79)	75 (80)	0.156
Black/African American	10 (7)	12 (13)	
Asian	8 (6)	1 (1)	
Others	11 (8)	6 (6)	
Ethnicity, Hispanic (%)	11 (8)	7 (8)	0.889
Body mass index, mean (SD)	20.0 (2.8)	20.6 (3.2)	0.248
Risser stage (%)			
0	4 (3)	0 (0)	0.003
1	7 (5)	3 (3)	
2	12 (9)	7 (8)	
3	25 (18)	5 (5)	
4	89 (65)	77 (82)	
5	0 (0)	2 (2)	
Spine height mm, mean (SD)	406.0 (24.9)	412.0 (23.8)	0.072
Total body height cm, mean (SD)	162.8 (7.7)	165.7 (8.6)	0.009
Follow-up months, mean (SD)	37.4 (15.8)	33.4 (16.8)	0.023

*SD* standard deviation, *SMS* Sanders maturation stage

**Table 2:** Baseline curve characteristics and brace treatment status

	SMS 7A	SMS 7B	*P* value
Major curve magnitude, mean (SD)	32.5 (7.9)	32.7 (8.2)	0.994
Major curve type, *n* (%)			
Thoracic	70 (51)	46 (49)	0.926
Lumbar/thoracolumbar	35 (26)	25 (27)	
Double major	21 (23)	23 (24)	
Family history of scoliosis (%)			
No	64 (47)	46 (49)	0.965
Yes	67 (49)	47 (50)	
Unknown	6 (4)	1 (1)	
Bracing status at SMS 7 (%)			
No/discontinuing brace	70 (51)	60 (64)	0.057
Weaning brace	56 (41)	32 (34)	
Continuing brace	11 (8)	2 (2)	

*SD* standard deviation, *SMS* Sanders maturation stage

**Fig. 2:**
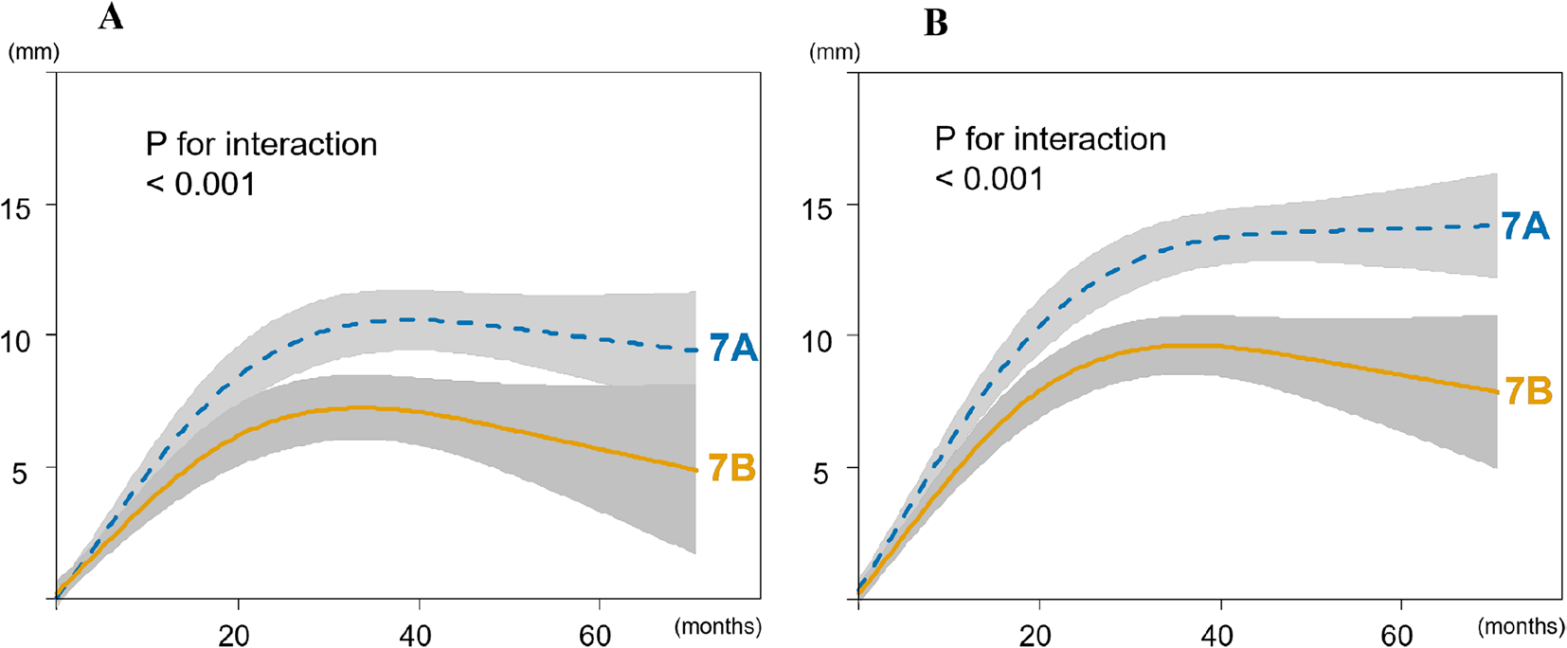
Association between spine height gain and observation periods adjusted for age, sex, body mass index, and race. The trajectory of spine height gain significantly differed between Sanders maturation stage 7A and 7B (*P* for interaction < 0.001) in both uncorrected (**A**) and corrected (**B**) data. The gray zone indicates 95% confidence intervals

**Fig. 3:**
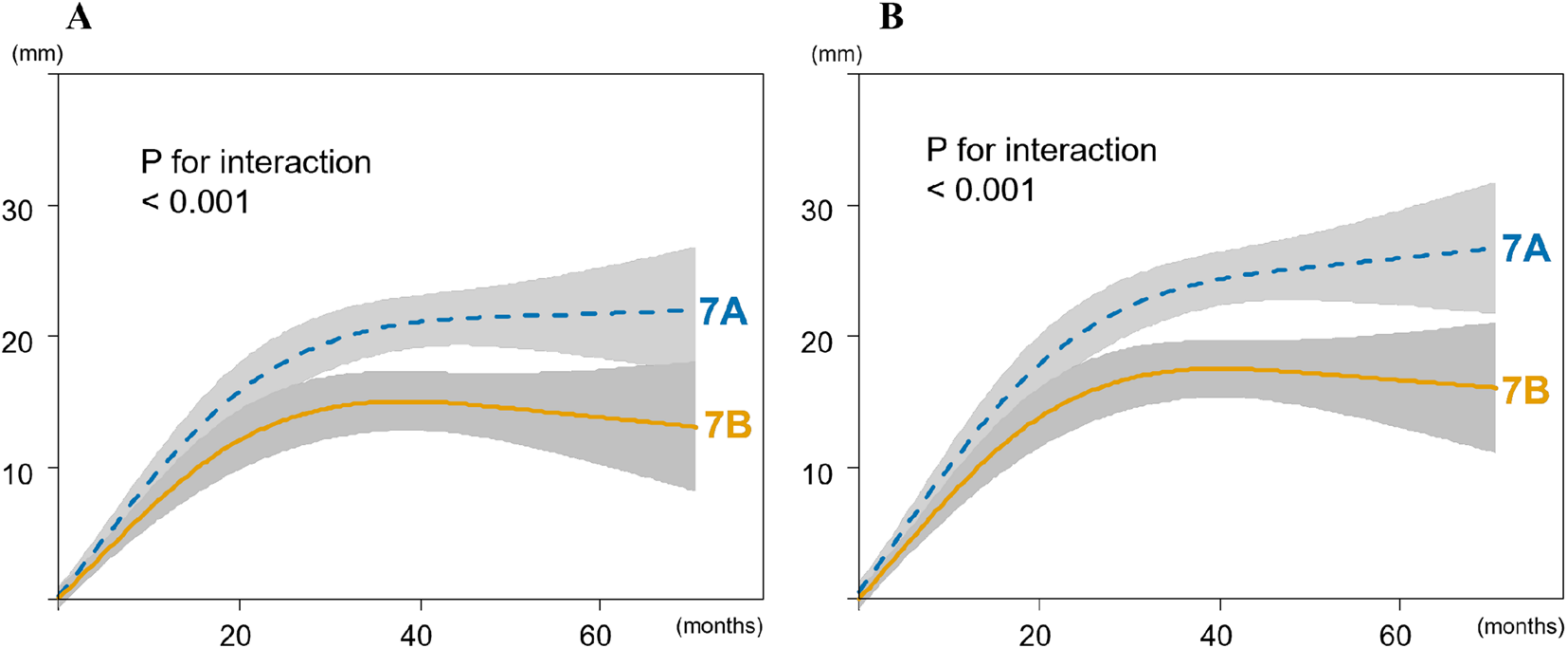
Association between total body height gain and observation periods adjusted for age, sex, body mass index, and race. The trajectory of total body height gain plateaued in the later phase (*P* for nonlinearity < 0.001), with a significant difference between Sanders maturation stage 7A and 7B (*P* for interaction < 0.001) in both uncorrected (**A**) and corrected (**B**) data. The gray zone indicates 95% confidence intervals

Spine height gain was significantly greater in SMS 7A than in 7B (9.9 ± 5.8 mm vs. 6.3 ± 5.3 mm, *P* < 0.001) indicating a modest growth advantage for SMS 7A with a mean difference of 3.6 mm. This difference became more pronounced after adjustment for curve magnitude (12.6 ± 5.6 mm vs. 8.4 ± 4.8 mm, *P* < 0.001). The multivariate non-linear regression analysis further highlighted a significant interaction between SMS 7 subtypes and observation periods (*P* for interaction < 0.001) indicating variations in growth trajectories. Specifically, patients classified as SMS 7A exhibited a greater spine height gain and reached the plateau phase later compared with those in the SMS 7B group (Fig. 2). For total body height gain, a similar pattern was observed. The gain was significantly greater in SMS 7A vs. 7B (19.8 ± 10.7 mm vs. 13.4 ± 10.6 mm, *P* < 0.001) with a mean difference of 6.6 mm. This disparity was further accentuated after adjusting for curve magnitude (22.5 ± 11.2 mm vs. 15.5 ± 10.4 mm, *P* < 0.001). The multivariate non-linear regression model showed that growth trajectory was significantly more pronounced in SMS 7A than 7B (*P* for interaction < 0.001) throughout the observational period (Fig. 3).

**Fig. 4:**
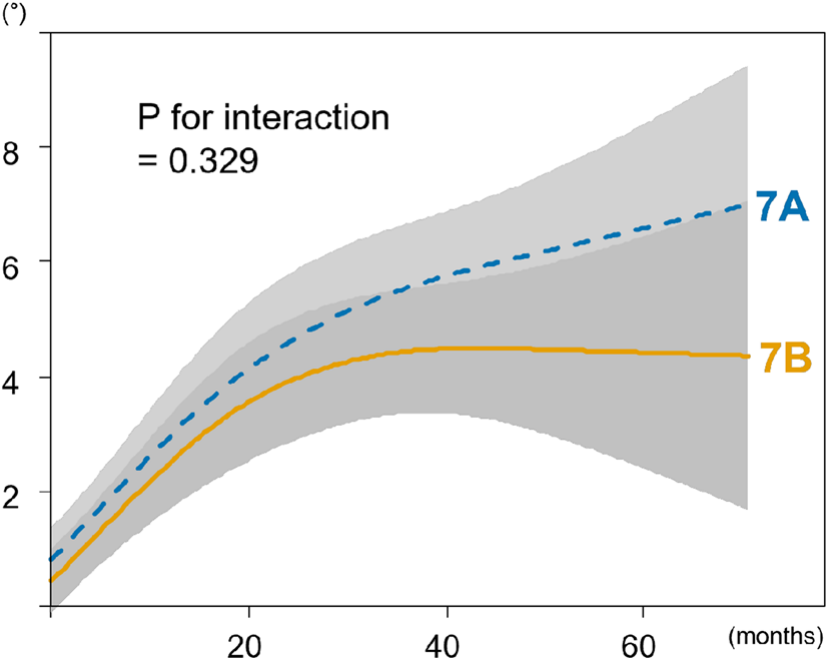
Association between scoliosis progression and observation periods adjusted for age, sex, body mass index, curve pattern, initial curve magnitude, and bracing status. The rate of scoliosis progression decreased in the later phase (*P* for nonlinearity = 0.001). There was no significant difference in scoliosis behavior between Sanders maturation stage 7A and 7B (*P* for interaction = 0.329). The gray zone indicates 95% confidence intervals

No significant difference was observed in curve progression between SMS 7A and 7B (4.8 ± 5.0° vs. 3.5 ± 4.2°, *P* = 0.103). According to the non-linear regression model, progression predominantly took place within the initial 2 years (Fig. 4). While SMS 7A looked as though gradual curve progression could be expected beyond this period, the variation was substantial and did not present any significant interaction (*P* = 0.329). More strikingly, curve progression exceeding 10° was considerably more frequent in SMS 7A than in 7B (18% vs. 7%, *P* = 0.020). In the logistic regression analysis, SMS 7A emerged as a significant risk factor associated with a curve progression of more than 10°, boasting an adjusted odds ratio of 3.31 (95% CI [1.31, 9.29], *P* = 0.015) (Table 3). Percentages of patients whose curve progressed to 50° or more are displayed in Table 4. No surgery was required for curves less than 30° at SMS 7, whereas curves exceeding 40° were likely to progress above 50° in both SMS 7A and 7B (68% versus 61%). In patients with 30° to 39° curves, SMS 7A patients had a higher percentage progressing to over 50° than those at 7B, although the difference was not significant (16% versus 5%, *P* = 0.124).

**Table 3:** Logistic regression model for predicting curve progression over 10°

	Adjusted OR	95% CI	*P* value
Age	1.08	0.68–1.72	0.746
Sex (boys)	1.09	0.27–4.16	0.904
Body mass index	0.98	0.84–1.12	0.742
Family history of scoliosis			
Yes	0.86	0.37–1.99	0.730
Unknown	0.68	0.03–6.01	0.751
Curve location (reference: double major)			
Lumbar/thoracolumbar	0.35	0.07–1.39	0.159
Thoracic	1.41	0.57–3.74	0.467
Major curve magnitude			
30° to 39°	9.53	2.48–63.3	0.004
≥ 40°	8.69	2.05–60.33	0.009
SMS 7A	3.31	1.31–9.29	0.015
Bracing status			
Weaning brace	0.49	0.19–1.22	0.136
Continuing brace	1.37	0.26–5.86	0.686
Follow-up periods (months)	1.01	0.99–1.04	0.223

*SMS* Sanders maturation stage, *OR* odds ratio

**Table 4:** Number and percentage of patients reaching surgical range (≥ 50°)

Baseline curve magnitude	SMS 7A	SMS 7B
	*n*/*N*, % (95% CI)	*n*/*N*, % (95% CI)
< 30°	0/46	0/31
	0% (0–7)	0% (0–11)
30° to 39°	10/63	2/39
	16% (8–27)	5% (1–17)
≥ 40°	19/28	14/24
	68% (48–84)	58% (37–78)

*SMS* Sanders maturation stage

## Discussion

SMS 7 is regarded as a hallmark of growth cessation and is crucial in marking the conclusion of treatment for patients in their growth phase. Sanders et al. [22, 23], by continuously monitoring the same individuals, established that growth patterns are mostly consistent, with heights at SMS 7 reaching 98–99% of final stature suggesting there is residual 1–2% height growth even at SMS 7. Grothaus et al. [17] observed an average height increase of 18 mm over 2 years in 89 patients with AIS who reached SMS 7. Our study is the first to showcase the clear differences in spine height and total body height growth between SMS 7A and 7B. Specifically, those categorized as 7A experienced growth of about 10 mm in spine height and 20 mm in total body height. In comparison, the 7B group saw growth of roughly 6 mm and 14 mm, respectively. While these differences might not seem large, they represented a significant difference in growth potential between 7A and 7B, even after adjusting for various confounders. The longitudinal findings further underscore this distinction, elucidating divergent growth trajectories inherent to each subtype. Specifically, patients classified SMS 7A might require more meticulous and prolonged observation. Such nuanced variations can be decisive for patients deliberating the continuation of their current treatment.

Previous studies have shed light on the behavior of curves at SMS 7, highlighting their potential for progression, particularly for those exceeding 40°. Grothaus et al. [17] documented an average 5° curve progression, with 12% of patients possessing curves over 40° advancing to the surgical threshold. Similarly, Johnson et al. [10] noted that 37% of SMS 7–8 patients with curves exceeding 40° progressed to the surgical range, a progression not observed in patients with curves below this magnitude. Cheung et al. [18] observed a 6-month progression of more than 5° in 11% of patients with curves less than 40° at SMS 7A compared with none at 7B. However, even at 7B, 21% of patients with a curve of 40° or more exhibited similar progression. The current study revealed a consistent curve progression of 4–5° for both 7A and 7B subtypes, with a notably higher risk of progression exceeding 10° in 7A, evidenced by an adjusted odds ratio of 3.1. Additionally, over half of patients with curves exceeding 40° progressed to surgery. Remarkably, even with curves of 30° to 39°, 16% of 7A and 5% of 7B patients advanced to the surgical threshold. These findings underscore the inherent risk of curve progression at SMS 7, especially in subtype 7A.

The noticeable higher growth potential in SMS 7A and significant curve progression risks necessitate a cautious approach toward brace discontinuation in this subgroup. Multiple studies have reported scoliosis progression post-brace removal, with more pronounced advancement in cases exhibiting larger curves and rotational deformities [24–26]. A recent review [27] highlighted a rapid progression rate of 0.8° per year shortly after brace weaning, which tapered to a mild 0.2° per year over the long term. Moreover, larger curves were notably more prone to progression. Our study parallels these observations indicating a steep curve progression within the first 2 years post SMS 7, which after that, moderates. Particularly, curves beyond 30° demonstrated a heightened risk of significant progression. Guidance on brace weaning methodology remains undefined, with existing guidelines suggesting a minimum weaning period of 6 months, albeit with a low evidence level of C [28]. No clear association between varying brace therapy strategies and substantial curve progression was noted in our study. Considering the patient distress deriving from an indiscriminate continuation of brace therapy [29], it is reasonable to discontinue bracing at SMS 7A for smaller curves below 30°, where the risks of substantial progression are minimal and the likelihood of necessitating surgery is exceedingly low. On the other hand, for larger curves, it would be more secure to discontinue the brace at SMS 7B, while the ability to thwart surgery in patients with curves exceeding 40° remains uncertain [18].

The Risser stage, particularly stages 4 and 5, is a widely accepted index for indicating skeletal maturity and concluding brace treatment. However, discrepancies between SMS and Risser stage have been reported in the literature. Minkara et al. [5] highlighted a 3.6% risk of mismatch for patients with a Risser stage of 0 and 1 and an SMS of 6 and 7. This mismatch could lead to unnecessarily prolonged treatment. Further complicating this issue, Neal et al. [6] reported that 23.2% of patients with an SMS of 7 had not yet reached a Risser stage of 4. The present study identifies a significant difference in Risser stage between SMS 7A and 7B, which reinforces the observed variability between SMS and Risser stages. Notably, 16% of patients with an SMS of 7B corresponded to a Risser stage of 1–3. This suggests that these patients could be exposed to a prolonged period of brace treatment. Given these findings, a detailed assessment of skeletal maturity incorporating the SMS 7 subclassification is recommended.

This study presents several limitations that warrant consideration. First, the retrospective nature of this study renders it susceptible to selection bias. Most patients diagnosed at SMS 7 were excluded from the study due to inadequate follow-up, thus the potential impacts of these exclusions remain unclear. Additionally, scoliosis progression at the baseline visit might affect the decision-making process for individual treatment. Second, the measurement of spinal height was conducted using 2D, not three-dimensional imaging, thereby potentially being influenced by spinal alignment. However, we employed a validated formula to obtain corrected height values accounting for the decrease in spinal height induced by scoliosis. Third, the follow-up duration varied among patients, particularly shorter in the SMS 7B group, likely due to more patients reaching Risser stage 5 within less than 2 years. Although some patients did not reach Risser stage 5, the findings suggest that a follow-up period exceeding 2 years would not significantly impact the outcomes. Moreover, the multivariate analysis has accounted for these variations in follow-up duration. Fourth, inherent to the retrospective nature of the study, there were differences in treatment strategies among patients, which could potentially affect curve progression. While multivariate analysis was conducted to account for these variations, information regarding brace compliance, daily duration of brace use, age at brace initiation, and the degree of in-brace correction was not evaluated. Last, most of the participants were Caucasian females. While the influence of sex, race, and ethnicity was considered in the multivariate analysis, it remains uncertain whether the results of this study can be universally applied to all patients with AIS worldwide. These limitations should be taken into account when interpreting the findings and their implications.

## Conclusion

This study elucidates a subtle yet significant discrepancy in spine height and total body height growth potential between SMS 7 subtypes, which persisted even when considering height reduction associated with scoliosis and other confounding factors. It was evident that a considerably larger growth potential was retained in SMS 7A, along with a higher risk of severe curve progression. These insights impart crucial implications for the concluding stages of treatment in growing children. It is reasonable to discontinue bracing at SMS 7A for AIS patients with smaller curves, yet for those with larger curves, continuing bracing until SMS 7B is a more prudent approach.

## Data Availability

Data are available upon reasonable request.
